# Brainstem neuroimaging of nociception and pain circuitries

**DOI:** 10.1097/PR9.0000000000000745

**Published:** 2019-08-07

**Authors:** Vitaly Napadow, Roberta Sclocco, Luke A. Henderson

**Affiliations:** a Department of Radiology, Athinoula A. Martinos Center for Biomedical Imaging, Massachusetts General Hospital, Harvard Medical School, Charlestown, MA, USA; b Department of Radiology, Logan University, Chesterfield, MO, USA; c Department of Anatomy and Histology, University of Sydney, Sydney, New South Wales, Australia

**Keywords:** Brainstem, Imaging, fMRI, Ultrahigh field, Periaqueductal gray, Rostroventromedial medulla, Diffuse noxious inhibitory control, Descending inhibition, Medulla, Pons, Midbrain

## Abstract

The brainstem is known to be an important brain area for nociception and pain processing, and both relaying and coordinating signaling between the cerebrum, cerebellum, and spinal cord. Although preclinical models of pain have characterized the many roles that brainstem nuclei play in nociceptive processing, the degree to which these circuitries extend to humans is not as well known. Unfortunately, the brainstem is also a very challenging region to evaluate in humans with neuroimaging. The challenges for human brainstem imaging arise from the location of this elongated brain structure, proximity to cardiorespiratory noise sources, and the size of its constituent nuclei. These challenges can require dedicated approaches to brainstem imaging, which should be adopted when study hypotheses are focused on brainstem processing of nociception or modulation of pain perception. In fact, our review will highlight many pain neuroimaging studies that have reported some brainstem involvement in nociceptive processing and chronic pain pathology. However, we note that with recent advances in neuroimaging leading to improved spatial and temporal resolution, more studies are needed that take advantage of data collection and analysis methods focused on the challenges of brainstem neuroimaging.

## 1. Introduction

The brainstem is a critical area for nociception and pain processing, as well as relaying and coordinating signaling between the cerebrum, cerebellum, and spinal cord. It is composed of 3 distinct subregions—the medulla (most caudal), pons, and midbrain (most cranial). Human functional magnetic resonance imaging (fMRI) studies have evaluated acute and chronic pain processing in the brainstem at both conventional (eg, 3 T) and ultrahigh-field (7 T and above) magnet strength, whereas structural MRI studies have assessed how gray matter volume and white matter integrity in the brainstem are associated with acute pain processing and are altered by chronic pain. Our review will highlight important challenges in brainstem neuroimaging (reviewed more in-depth elsewhere^108^), particularly for nuclei purported to be linked with pain processing. We will also provide promising examples of research demonstrating progress in human brainstem imaging to better understand the encoding of nociception and pain.

## 2. Brainstem nuclei involved in nociception and pain processing

**Figure 1.**
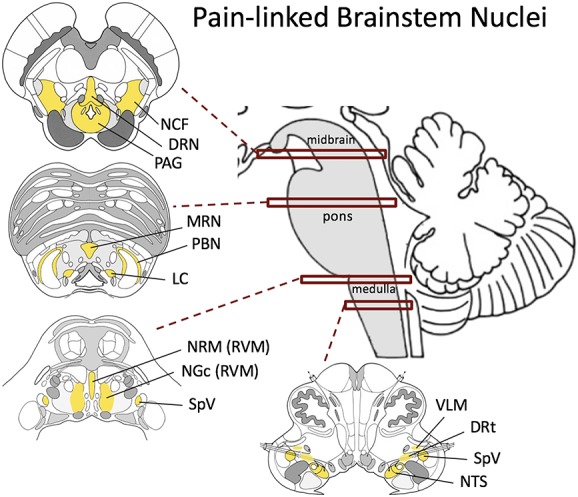
Schematic of brainstem nuclei linked with pain processing. DRN, dorsal raphe nucleus; DRt, dorsal reticular nucleus; LC, locus coeruleus; MRN, median raphe nucleus; NCF, nucleus cuneiformis; NGc, nucleus gigantocellularis; NRM, nucleus raphe magnus; NTS, nucleus tractus solitarii; PAG, periaqueductal gray; PBN, parabrachial nucleus; RVM, rostral ventromedial medulla; SpV, spinal trigeminal nucleus; VLM, ventrolateral medulla.

Nociception is defined as the neural processes of encoding harmful stimuli,^72^ and the brainstem plays a cardinal role in both nociception and acute pain processing. In fact, most animal studies of pain processing have been focused on nociceptive encoding and have contributed heavily to our mechanistic understanding of the brainstem's role in pain processing. Multiple brainstem nuclei are known to be involved in pain processing, and include the periaqueductal gray (PAG) and nucleus cuneiformis (NCF) in the midbrain; dorsal raphe nuclei and median raphe nuclei, parabrachial nucleus, and locus coeruleus (LC) in the pons; and rostral ventromedial medulla (RVM, composed mainly of nucleus reticularis gigantocellularis (NGc) and nucleus raphe magnus), ventrolateral medulla (VLM), and dorsal reticular nucleus (DRt) in the medulla (Fig. 1). In addition, nociceptive input from the face and viscera enters the medulla and terminates in the spinal trigeminal nucleus (SpV) and nucleus tractus solitarii (NTS).^29,103^ These sensory nuclei also play an important role in both nociception and acute/chronic pain processing.

The PAG is a key region for pain processing. Although simple withdrawal responses to acute noxious stimuli are organized at the level of the spinal cord (or analogous brainstem nuclei for craniofacial reflexes), more complex and arguably more critical behavioral responses to noxious stimuli are integrated in higher brainstem regions such as in the PAG. The PAG surrounds the mesencephalic aqueduct and is composed of several columns with neurons associated with different neurotransmitter systems. The PAG has no clear cytoarchitectonic boundaries. However, distinct behavioral, cardiovascular, and afferent and efferent connection patterns have resulted in the PAG being divided into 4 longitudinal columns along the aqueduct, the dorsomedial PAG (dmPAG), dorsolateral PAG (dlPAG), lateral PAG (lPAG), and ventrolateral PAG (vlPAG) subdivisions.^60^ Active coping behavioral responses to pain that are critical for survival such as fight and flight can be evoked by direct stimulation of the lPAG, whereas passive coping behaviors such as quiescence are mediated by the vlPAG.^6^ In addition to the motor responses characterizing active and passive behaviors, direct PAG stimulation alters arterial pressure, heart rate, and blood flow patterns to support these behaviors. Stimulation also modulates vigilance and reactivity, and all of these behavioral/physiological responses are coupled to a powerful analgesia. While the expression of these behaviors can be modulated by afferent inputs to the PAG from higher brain regions such as the dorsomedial/orbital prefrontal cortex, cingulate cortex, and central nucleus of the amygdala,^5^ the PAG itself contains all the neural hardware required to produce these integrated defensive behaviours.^60^ Furthermore, nociceptive inputs to the lPAG are arranged somatotopically with SpV projections terminating in the rostral lPAG, and cervical and lumbar spinal projections at progressively more caudal levels.^10,126^ In addition, somatic and visceral nociceptive inputs are column specific, with afference from superficial nociceptors conveyed to lPAG and dlPAG columns (mainly through A-delta fibers), whereas the vlPAG receives afference from muscle/fascia and cutaneous C-fiber nociceptors, as well as visceral afference through the NTS.^10,98^ Given the fundamental nature of these animal model–derived behavioral responses, it is assumed that the circuitry responsible for them is preserved in humans, although this is yet to be definitively established.

As described above, PAG stimulation can produce a powerful analgesia, inhibiting incoming noxious information at the dorsal horn and SpV through PAG projections to the RVM.^25,44,51^ The PAG is heavily interconnected with the RVM, which is composed of so-called ON, OFF, and NEUTRAL classes of neurons within the NGc and nucleus raphe magnus, which can both facilitate and inhibit incoming noxious information. OFF-cells are silent during nociceptive input^43^ and were found to inhibit incoming nociceptive inputs.^56^ Alternatively, pain facilitation and behavioral hyperalgesia have been associated with increased activity of RVM ON-cells.^45^ Although the analgesia that occurs in concert with active and passive behaviors likely aids the individual in avoiding threatening situations, PAG–RVM pathways supporting descending pain inhibition also appear to be critical in mediating “higher-order” analgesic responses such as stress-induced analgesia. Periaqueductal gray–RVM pathways supporting pain facilitation^132^ through RVM ON-cells have been proposed to underlie some chronic pain conditions, manifested by increased PAG–RVM response during evoked noxious input.^97^ However, with respect to human neuroimaging, preclinical studies have clearly shown that the various RVM cell types are anatomically intermingled, and thus, activity of individual populations cannot be separated with typical fMRI approaches in humans.

In addition to the well-described PAG–RVM pain modulatory system, other brainstem nuclei have been implicated in nociceptive input regulation. These include 2 regions in the medulla, the DRt and caudal VLM.^46,48,70^ The DRt is critical for conditioned pain modulation, an analgesic response whereby central nervous system response to one noxious stimulus is inhibited by the application of a second noxious stimulus, and acts by inhibiting nociceptive inputs within the spinal and medullary (ie, SpV) dorsal horns. By contrast, the presence of documented ON- and OFF-cells in the VLM points to a dual inhibitory/facilitatory role, similar to the RVM.^99^ In the pons, the LC is a source for noradrenergic input to the brain, regulating attention and arousal^104^ but also highly involved in pain processing, such as with distraction analgesia. Optogenetic activation of different subpopulations of LC neurons has been shown to exert both pronociceptive and antinociceptive effects in rats, suggesting a bidirectional influence of this nucleus.^57^ In the midbrain, the NCF is also involved in both ascending transmission and modulation of nociceptive afference. Importantly, the NCF also contains ON-/OFF-cells^55^ and, like the PAG, projects to the RVM. In fact, its proximity to the PAG (the NCF is ventral and lateral to the PAG) has led some authors to mistakenly attribute NCF fMRI response to the PAG.

Although these brainstem nuclei can clearly alter incoming nociceptive information, more research is needed to delineate the roles they play in the initiation and/or maintenance of various chronic pain conditions in humans.

## 3. Challenges of brainstem neuroimaging

Preclinical studies have been successful at defining extremely small groups of neurons within the brainstem responsible for discrete functions, and it is assumed that the relatively primitive functions of these brainstem nuclei are preserved across species. Noninvasive neuroimaging is a powerful tool to evaluate brain activity in humans. However, the challenges for human brainstem imaging are many and arise from the location of this elongated brain structure, its proximity to cardiorespiratory noise sources, and the size of its constituent nuclei. These challenges can require dedicated approaches to brainstem imaging, which should be adopted when study hypotheses are focused on brainstem processing of nociception or modulation of pain perception.

**Figure 2.**
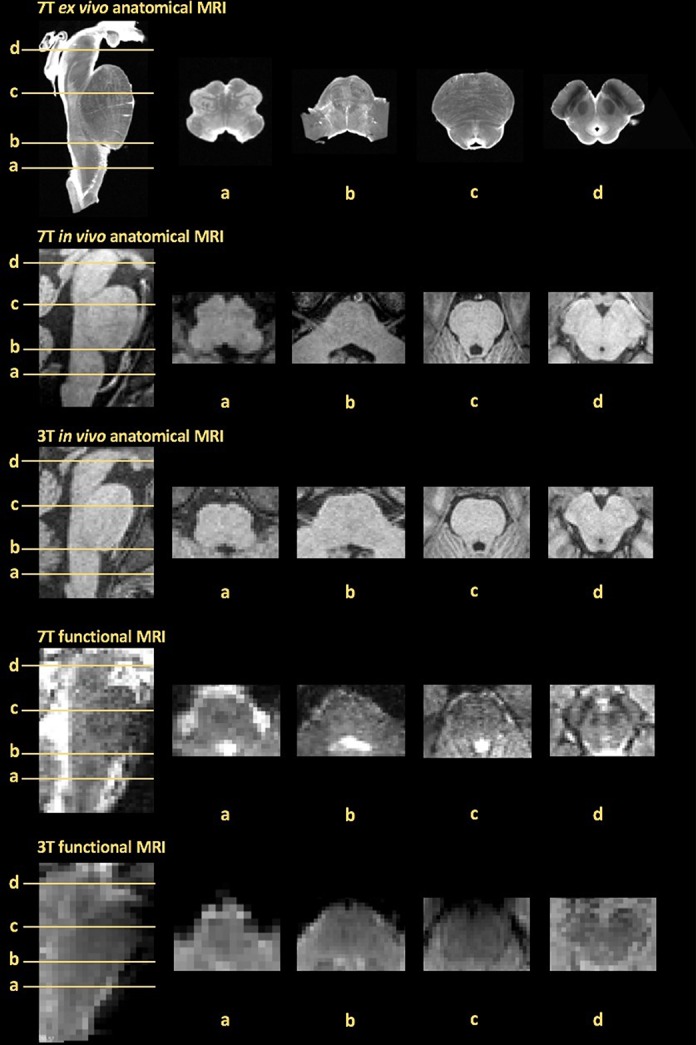
Representative examples of anatomical and functional brainstem MRI data quality obtained at different magnetic field strengths (7 vs 3 T). Axial slices (a–d) include pain-associated brainstem nuclei of interest from Figure 1. From the top: ex vivo anatomical obtained at 7 T (0.2-mm isotropic voxels, B0 image from DTI acquisition) generously provided by the laboratory of Dr. Alan Johnson^23^; in vivo anatomical obtained at 7 T (0.75-mm isotropic voxels, Multi-Echo MPRAGE); in vivo anatomical obtained at 3 T (1-mm isotropic voxels, Multi-Echo MPRAGE); functional MRI data obtained at 7 T (1.2-mm isotropic voxels, TR = 0.99 seconds, TE = 23 ms, phase-encoding R-L, Simultaneous Multi Slice, SMS factor = 2); and functional MRI data obtained at 3 T (2-mm isotropic voxels, TR = 1.25 seconds, TE = 33 ms, phase-encoding A-P, SMS factor = 5). DTI, diffusion tensor imaging; MRI, magnetic resonance imaging; TR, repetition time; TE, echo time.

First, many brainstem nuclei are elongated, with an average cross-sectional diameter of only a few millimeters or less in humans, and are considerably smaller than our current delineations of regional functional specializations in higher cortical and subcortical structures.^1^ The typical in-plane fMRI spatial resolution is 2 to 4 mm at 3 T, whereas ultrahigh-field (7 T and above) fMRI spatial resolution can be on the order of 1 mm isotropic (Fig. 2).^12,14,26,32,42,100,105,109,123^ Multiband acceleration can significantly improve spatiotemporal resolution for echo planar imaging fMRI, both at 3 and 7 T. However, caution should be used with very high acceleration factors because of potentially reduced signal-to-noise ratio at high acceleration factors. This has been noted for resting-state connectivity^101^ and task-evoked fMRI metrics, which early reports suggest may not benefit from the higher temporal resolution afforded by multiband fMRI.^33^ How multiband imaging specifically impacts the brainstem fMRI signal is not well understood, and parameters such as slice orientation and slice thickness can significantly impact the upper limits on acceleration factors. Regardless of which acceleration factor is chosen, the relatively limited spatial resolution for most fMRI methods poses a major challenge for imaging brainstem function in humans, and some of the standard analysis procedures used when exploring activity changes in higher-order brain regions are less appropriate when investigating brainstem function. From an analysis perspective, owing to the small cross-sectional area of most brainstem nuclei, correction for multiple comparisons should use voxel-based correction approaches, as opposed to cluster-based approaches that are skewed to larger activation clusters. In fact, cluster correction approaches typically used for whole-brain imaging will, many times, identify brainstem clusters only when such clusters cover high-noise cerebrospinal fluid or blood vessel voxels surrounding the brainstem parenchyma. Furthermore, nonparametric approaches (eg, permutation testing) or Bayesian statistics may be preferred over parametric general linear models, as smoothness assumptions associated with the latter are often not met in the brainstem.^8^

Physiological (ie, cardiorespiratory) noise sources can significantly degrade the MRI signal. Such noise can stem from magnetic field changes due to chest motion (off-resonance B_0_ effects present in both functional and structural MRI), as well as from the propagation of cardiac and respiratory pulse pressure waves in arteries, cerebrospinal fluid spaces, and parenchyma. Moreover, compared with higher brain regions, caudal brainstem locations are closer in proximity to noise-generating sources such as the heart and lungs,^19^ potentially increasing the level of cardiorespiratory noise. Although fMRI physiological noise increases with field strength,^66,117^ its contribution is mitigated by decreasing voxel size,^15^ a strategy commonly used in ultrahigh-field fMRI. Many pain-processing brainstem regions lie close to areas of high susceptibility as evident in unmasked brainstem fMRI data using independent component analyses.^9^ Hence, some authors have advocated a number of techniques to limit these potential artefacts such as restricting fMRI brainstem analyses to an anatomically defined tight brainstem mask,^7,82,109^ applied *before* any spatial smoothing, thus limiting the extension of cardiorespiratory physiological noise near the surface from corrupting the fMRI signal from deeper brainstem nuclei. In addition, chest motion effects can be partially compensated by retrospective physiological noise correction strategies, which use physiological recordings for cardiac and respiratory frequency band estimates^19,50^ or respiratory-related information extracted from the image phase.^13^

In addition to potential physiological noise, brainstem MRI is plagued by magnetic susceptibility–induced distortions because of its proximity to air-filled cavities and the steep magnetic susceptibility gradient produced by the air–tissue boundary.^49^ Although similar susceptibilities occur in higher brain regions such as the prefrontal cortex (due to the frontal air sinus) and the temporal lobe (due to the mastoid air cells)—for the brainstem, such distortions can hamper coregistration and transformation to a standard space template. Furthermore, the brainstem is located in a narrow bony canal, which narrows as it extends caudally, leading to further susceptibility-induced distortions in caudal regions due to this bone–tissue proximity. Solutions have included the use of an anatomical reference data set with identical distortion to the BOLD fMRI data, applied both at 3 and 7 T,^52,102,116^ enabling improved masking of brainstem structures by transforming a brainstem mask defined in standard space to individual functional space^109^ and the use of a brainstem isolation and an anatomical specific template.^38^

Another challenge for brainstem imaging stems from a lack of dedicated, comprehensive probabilistic brainstem atlas that includes the large number of nuclei across the midbrain, pons, and medulla. Although existing atlases^34,37,62,118^ released with the common neuroimaging software (eg, FSL, FreeSurfer, and SPM) include several cortical and subcortical regions, most brainstem nuclei and their subdivisions are not available. Atlases including a limited number of brainstem nuclei, such as the substantia nigra, red nucleus, and subthalamic nucleus,^28,62,67,78,84^ are available, although their subdivisions are not. Recently, Mori et al. showed the feasibility of ex vivo diffusion tensor imaging (DTI) of several nuclei important for motor and cranial nerve functional systems in a single postmortem brainstem specimen.^2^ However, further attempts are ongoing, particularly at 7 T,^11,12^ and in the future, a dedicated probabilistic atlas for a larger number of brainstem nuclei in a standard space will greatly enhance localization. Given the difficulty in localization for many brainstem nuclei, it is recommended that imaging results be shown with axial slices from a tilted (ie, pitched along a medial–lateral axis in the sagittal plane) brainstem underlay, to match existing published atlases.^22,91^

Difficulty in brainstem atlas creation also extends to the copious white matter and densely crossing fiber tracts that pass through this brain region. Fiber crossings and decussations for major white matter tracts create challenges for accurate 3D determination of the pathways that interconnect different brainstem nuclei, and connect the spinal cord and brainstem to higher cortical structures. Spatial resolution is an important consideration for white matter tracking as well, and Mori et al. developed and publicly released an ex vivo DTI atlas of brainstem white matter tracts.^84,85^ Further efforts are ongoing to better account for microstructure and crossing fibers in the brainstem, and a recently published brainstem white matter atlas, based on DTI data from the publicly released Human Connectome Project database, delineated 23 main brainstem white matter bundles covering motor and sensory tracts, as well as the cerebellar peduncles.^114^

Ultimately, improved spatial resolution provided by high-field MRI will provide the basis for improved exploration of regional brainstem function. For instance, Satpute and coauthors used ultrahigh-field (7 T) fMRI to image the PAG with a 0.75-mm isotropic spatial resolution,^105^ while exposing participants to emotionally aversive images. Activation was localized to the lPAG and dmPAG rostrally, and to the vlPAG caudally, consistent with observations from animal studies,^87,88^ and further supporting the feasibility of exploring the functional architecture of small, difficult to image brainstem nuclei with ultrahigh-field fMRI.

## 4. Brainstem neuroimaging for nociception and acute pain processing

Neuroimaging studies have attempted to extend preclinical animal research, which has mostly focused on neural networks responsible for acute pain processing. Early brainstem-focused fMRI studies were able to demonstrate activation in brainstem nuclei such as PAG, NCF, ventral tegmental area, substantia nigra, and dorsolateral pons (ie, parabrachial nucleus and LC) in response to both somatic cutaneous and visceral (eg, rectal) nociceptive stimuli.^40^ In addition, the orofacial system provides a unique opportunity to explore pain processing at the primary afferent synapse in the SpV, located in the medulla and caudal pons. A number of studies have shown activation of the SpV during acute cutaneous and muscle noxious stimuli applied to the orofacial region,^16,31,68,94,131^ supporting the well-characterized pathways described by animal research.^25^ An important lesson from such studies is that adequate spatial resolution and functional/structural coregistration are critical for robustly determining fMRI response in elongated, small cross-sectional area nuclei such as the SpV and NTS in the medulla and pons. Thus, data collection and analysis methods for brainstem imaging may need to deviate from conventional cortical imaging approaches.

In addition, acute pain fMRI studies were recently extended to assess brainstem circuitry responsible for the nociceptive processing phenomena of temporal summation and conditioned pain modulation. A recent brain imaging study used the application of a noxious muscle stimulus to the leg to inhibit orofacial acute pain, ie, conditioned pain modulation. They revealed that conditioned pain modulation is associated with reduced fMRI signal response in the DRt and dorsolateral pons, as well as the brainstem region receiving noxious orofacial afferents, ie, SpV.^129^ Reduced conditioned pain modulation responsiveness has been linked to chronic pain, underscoring the importance of further neuroimaging research on this phenomenon. Furthermore, a recent brain imaging study has linked activity in the PAG–RVM axis with temporal summation of pain,^17^ a phenomenon related to nociception wind-up in animal models of chronic pain and in individuals suffering from chronic pain.^96,120^

Although we have described the spatial resolution limitations of brainstem imaging, temporal resolution is also a limitation. Most functional brain imaging protocols collect a volume every 2 or more seconds, which effectively limits the temporal range of pain processing that can be explored. For example, temporal summation of pain requires a stimulus frequency of approximately 1 Hz, which is typically above the temporal resolution of fMRI, impacting event separability due to ambiguity in the delayed hemodynamic response function. Higher field strength scanners can allow for subsecond acquisition time frames. However, cruder forms of time-resolved brainstem responses during painful stimuli have been previously assessed with lower field fMRI. For example, instead of simple averaging over multiple repetitions of an evoked pain stimulus, independent modeling of each serial block (or event) from typical fMRI study designs allows for the assessment of temporal variations in brainstem activation.^92^ This approach was used to resolve nonlinear (eg, U-shaped) midbrain activation to cuff pain stimuli across the pain intensity spectrum^73^ and dishabituation phenomena for patients with chronic pain (see section 5.2 below). Brainstem processing may in fact be a major determinant for temporally variable pain perception with repeated nociceptive stimuli, and future studies should apply advanced neuroimaging analysis approaches to better assess time-resolved brainstem response to evoked pain stimuli.

It is important to note that although the brainstem pain-modulating circuits described above can modulate incoming nociceptive input at the primary afferent synapse, it is thought that higher brain areas can also modulate pain by influencing these brainstem circuits. For instance, a number of human neuroimaging studies have begun to evaluate how higher cognitive functions can modulate such circuitry. For example, Keltner et al.^61^ linked cognitive expectancy modulation of pain intensity with NCF responses during nociceptive stimuli, supporting the importance of brainstem mechanisms for cognitive modulation of pain. Similarly, Brooks et al.^18^ found that when high cognitive load reduced thermal pain ratings, there was a concomitant temperature × task interaction in LC fMRI response. In addition, Tinnermann et al.^115^ recently noted that value information (eg, “expensive” vs “cheap” pain cream) can upregulate nocebo hyperalgesia, an effect mediated by pregenual anterior cingulate cortex responses to heat pain stimuli and its connectivity to the vlPAG, which was also more activated during the high value nocebo condition. In fact, the measure of functional connectivity, ie, the strength of signal covariation between different brain regions, has also been commonly used to assess communication between brainstem and higher telencephalic regions in both resting and pain-processing studies. For instance, a recent 3 T fMRI study using resting-state fMRI revealed connections between the vlPAG and brain regions associated with descending pain modulation (anterior cingulate cortex, dorsal pons, and medulla), whereas the lPAG and dlPAG were connected with brain regions implicated in cognitive/executive functions (eg, middle frontal gyrus).^30^

In fact, given the critical role for the PAG in pain behaviors revealed in experimental animal investigations,^25^ PAG fMRI activity has been a common focus for many acute pain neuroimaging studies, although thus far mainly at conventional field strengths such as 3 T.^71^ As we noted the well-described fine parcellation of the PAG, it is important that future research is aimed at exploring this structure in greater detail using higher field strength scanners. Indeed, Hahn et al. compared fMRI responses to painful vs innocuous electrical stimulation at 3 and 7 T,^53^ adopting similar in-plane resolutions for both field strengths (1.48 × 1.48 mm^2^ at 3 T and 1.5 × 1.5 mm^2^ at 7 T), and found that PAG activation for painful vs innocuous stimulation was found only with 7 T fMRI, likely due to increased BOLD signal-to-noise ratio at higher field strengths. These results support an expanded role for ultrahigh-field fMRI in evaluating brainstem nociceptive circuitries, where spatial and temporal resolution can better target discrete brainstem nuclei such as the subdivisions of PAG, NCF, and the medullary components (RVM and VLM) of the descending pain modulatory system.

Of course, in addition to the sensory perceptual aspect of acute pain processing, noxious stimuli are often coupled to autonomic changes, and it is well known from extensive experimental animal investigations that autonomic nervous system activity is closely tied to pain perception. In addition to known nociception processing nuclei, the brainstem also contains sympathetic and parasympathetic premotor nuclei, some of which overlap with the pain modulation circuitry noted above. In fact, a recent 7 T fMRI study found that sustained (6 minutes) experimental pain reduced cardiovagal modulation (high-frequency heart rate variability [HF-HRV]), and brainstem nuclei associated with this pain-evoked HF-HRV reduction included RVM, ventral nucleus reticularis (Rt)/nucleus ambiguus, dorsal motor nucleus of the vagus/NTS, and LC.^109^ Such studies, combining high spatial resolution fMRI and high temporal resolution HF-HRV data, hold promise for multimodal imaging of brainstem circuitries supporting pain-associated autonomic responses, which have been shown to contribute to biomarker development for clinical pain perception.^69^

## 5. Brainstem neuroimaging for assessment of chronic pain mechanisms

The significant involvement of the brainstem in nociceptive processing also makes this brain region a likely key contributor to the pathophysiology of many chronic pain conditions. Chronic pain can develop after injury to the central nervous system above the level of the brainstem, such as that following thalamic stroke.^64^ However, it has been shown that even chronic pain conditions that involve injury to peripheral structures are characterized by changes in pain modulatory regions located within the brainstem.^20,80,122^ Indeed, brainstem imaging for chronic pain has been mainly applied on clinical pain disorders associated with cranial sensory nerves that enter the pons and medulla, such as trigeminal neuralgia, trigeminal neuropathy, and migraine. Notably, investigations in other chronic pain disorders have also begun to explore aberrant brainstem processing, particularly related to the PAG–RVM descending pain modulatory system.

### 5.1. Trigeminal neuralgia

Trigeminal neuralgia is a neuropathic pain disorder with high morbidity and is thought to arise from neurovascular compression of the trigeminal nerve at the root entry zone, within the pontine cistern.^74^ Although gross neurovascular compression is not always evident with standard clinical MRI, in a recent brainstem-focused study, DTI acquired with 1-mm in-plane resolution was used to assess white matter microstructure in the trigeminal nerve rootlets.^36^ Trigeminal neuralgia patients demonstrated lower fractional anisotropy in the affected (ipsilateral to the pain) trigeminal nerve. Fractional anisotropy is a DTI marker linked with white matter integrity, and a lower value in peripheral nerves, in conjunction with increased radial and mean diffusivity, suggests the existence of neuroinflammation and/or edema. This same group later found that effective surgical therapy reversed these DTI abnormalities, and change scores were correlated with pain relief.^35^ Such studies represent a nice example of a brainstem-linked chronic pain pathology, imaged with appropriate spatial resolution to assess hypothesis-driven brainstem-related morphological alterations, which are then linked to clinical outcomes after therapy. Interestingly, another trigeminal neuralgia DTI study suggested that fractional anisotropy may instead be elevated within brainstem areas consistent with the SpV.^124^ Future studies will be needed to corroborate and reconcile the few structural imaging findings that have been reported for this chronic pain population, and to extend these observations to better understand the functional plasticity associated with structural changes in trigeminal neuralgia. Although only few fMRI studies have evaluated functional alterations in brainstem circuitry associated with trigeminal neuralgia, a previous study did find that allodynia at the cutaneous trigger zone was associated with greater SpV activation.^83^

### 5.2. Migraine

Migraine is a neurovascular disorder characterized by altered neural processing in the central nervous system.^3,107,111^ Importantly, hyperalgesia, allodynia, and impaired habituation have been commonly reported in patients with migraine, even during the interictal phase (between attacks),^21^ suggesting impaired brainstem pain modulation circuitry. In fact, imaging studies have demonstrated that patients with migraine show interictal abnormalities in subcortical and brainstem regions including PAG, dorsal pons, and SpV, as well as activation of the dorsal pons and PAG during the migraine attack itself.^3,54,75,89,90^ These studies support the hypothesis that the PAG–RVM axis, which mediates descending inhibition and facilitation, is likely altered during migraine. Indeed, a recent fMRI study found that interictal migraineurs demonstrate reduced PAG activation in response to orofacial heat pain stimulation but enhanced resting PAG/RVM connectivity during evoked pain using a psychophysiological interaction analysis.^77^ In this same study, migraineurs also displayed greater pain-evoked activation in the SpV and reduced resting SpV/RVM connectivity. Another recent fMRI study found that although SpV response to innocuous trigeminosensory stimuli in interictal migraine patients was not greater than healthy adults, the transfer of information from the SpV was actually amplified in higher cortical regions such as hypothalamus and posterior insula (ie, elevated ratio of fMRI response in hypothalamus/insula vs SpV fMRI response), an effect modulated by patients' relative interictal phase.^68^ In fact, several recent studies have highlighted differential brain processing during the period immediately before a migraine attack relative to the interictal phase. Immediately before a migraine, there is increased SpV responses to innocuous trigeminosensory stimulation,^68^ increased Sp5 connectivity to the hypothalamus during noxious trigeminal stimulation,^106^ and increased amplitude of low-frequency oscillations in the resting fMRI signal for several brainstem regions including PAG and SpV.^79^

In addition to such functional brainstem neuroimaging studies, other forms of imaging have implicated important brain structural and functional changes in migraineurs. For instance, a recent positron emission tomography study with [^11^C]PBR28, a radioligand that binds to the 18 kDa translocator protein (TSPO) which is a marker of glial activation, noted elevated SpV uptake in migraineurs with aura.^4^ Structural T1-weighted MRI data have been used to evaluate deformable mesh models of different brainstem regions allowing for shape analyses in migraineurs. Using this technique, it was found that outward deformations in the lateral medulla and dorsolateral pons occur in migraineurs,^27^ implicating regions containing nuclei such as SpV in migraine pathophysiology. Furthermore, brainstem DTI studies in migraine have shown altered diffusivity in the PAG, RVM, and SpV in migraineurs,^76^ again implicating altered descending pain modulatory circuits in the pathophysiology of migraine. Future studies are warranted, particularly with improved spatial resolution and focusing on trigeminal nerve rootlets entering the pontomedullary junction, given the trigeminal entry zone DTI studies noted above for trigeminal neuralgia.

### 5.3. Temporomandibular disorder

Temporomandibular disorder (TMD) is a relatively common craniofacial pain disorder characterized by (mainly) myofascial pain within the temporomandibular joint and/or masticatory muscles adjacent to this joint. In TMD, nociceptive afference is directed to the brainstem along the trigeminal nerve, and DTI studies have found reduced fractional anisotropy and increased mean diffusivity and radial diffusivity in the trigeminal nerve roots entering the pons.^81^ These DTI changes were subsequently corroborated by another group,^125^ strongly suggesting that although gross abnormalities in peripheral nerve anatomy are not present for this idiopathic chronic pain disorder, microstructural changes along the trigeminal nerve can indeed be found. Structural MRI studies using voxel-based morphometry have also found altered SpV gray matter volume in patients with TMD, both increased^127^ and decreased,^125^ relative to healthy controls—a discrepancy that needs further research. Although BOLD fMRI assessments for brainstem processing in TMD have been scarce, an arterial spin labeling study assessed regional cerebral blood flow for patients with TMD and found that patients show increased blood flow in SpV compared with healthy controls, suggesting that painful TMD may be maintained by sustained activation of peripheral nociceptors in the temporomandibular joint and/or adjacent masticatory muscles.^128^ In summary, structural MRI studies point to altered neuroanatomy in brainstem structures primary to trigeminal pain processing. Functional MRI assessment of altered brainstem neurophysiology in TMD needs more research attention, although promising evidence has linked SpV physiology with TMD. Future research using dedicated brainstem fMRI methods is needed to further probe altered brainstem neurophysiology for this disorder.

### 5.4. Mechanisms of therapeutic interventions for chronic pain

Finally, it should be noted that brainstem imaging has also been used to assess therapeutic mechanisms. For instance, deep brain stimulation (DBS) of the PAG and periventricular gray has been applied for chronic pain, although the mechanisms of action are not completely understood. A recent positron emission tomography (PET) study used [^11^C]diprenorphine (DPN, an opioid radioligand) in a small cohort of patients with implanted PAG/periventricular gray DBS systems demonstrated decreased [^11^C]DPN binding in the caudal and dorsal PAG following DBS in the rostral dlPAG, suggesting a focal release of opioid peptides.^110^ While PET spatial resolution is more limited, we should note that ultrahigh-field MRI may indeed play an important role in presurgical planning and MR-guided surgery for precise lead placement,^24^ although clinical benefits of 7 T MRI have yet to be seen for DBS of the brainstem.^119^

Noninvasive neuromodulatory approaches for pain have also targeted brainstem nuclei by electrically stimulating cranial nerve innervated territories. One promising approach is the targeting of the NTS with transcutaneous vagus nerve stimulation (tVNS). Vagus nerve stimulation, which involves surgical placement of electrodes coiled around the cervical vagus nerve within the carotid sheath, has demonstrated efficacy for multiple disorders (eg, epilepsy and depression) and, recently, migraine.^58,86,130^ Despite the therapeutic potential of VNS, adverse events and complications associated with surgery and chronic stimulation limit broad applicability.^41^ Importantly, the NTS and SpV also receive somatosensory afference through the auricular branch of the vagus nerve (ABVN).^63,95^ Noninvasive (transcutaneous) methods of ABVN stimulation (tVNS) have been proposed,^121^ and preliminary 3 T neuroimaging studies have found that tVNS modulates brainstem and cortical areas similar to classical VNS,^39,65^ although a clinical trial suggested that tVNS may also reduce the frequency of migraine episodes.^112^ Interestingly, the dorsal medullary vagal system operates in tune with respiration and ABVN stimulation gated to exhalation may enhance tVNS outcomes for pain.^93^ Other studies have demonstrated that respiratory-gated tVNS can also enhance targeting of specific brainstem nuclei such as NTS, as recently shown for patients suffering from migraine.^47^ In fact, NTS response to auricular tVNS may also benefit from a more focal identification, by applying ultrahigh-field fMRI. Furthermore, future brainstem neuroimaging studies could use cranial nerve stimulation techniques such as tVNS (known to target distinct medullary nuclei) to optimize stimulation parameters for enhanced therapeutic response and, from a methodological point of view, to improve fMRI pulse sequences and analysis approaches for brainstem neuroimaging applications.

## 6. Conclusions and future directions

The brainstem is a critical structure for nociception and pain processing, both for acute experimental pain and chronic pain pathology. Unfortunately, the brainstem is also a very challenging region to evaluate in humans with neuroimaging. Many previous pain neuroimaging studies have reported some brainstem involvement in nociceptive processing and chronic pain pathology. However, most of these studies have been designed to assess cortical and/or supra-brainstem morphology and physiology, with only serendipitous brainstem findings reported when evident.

With recent advances in multiband accelerated neuroimaging leading to improved spatial and temporal resolution, more brainstem-focused studies are needed with modified data collection and analysis methods, taking into account the unique location of this brain region and relatively small size of many brainstem nuclei, compared with typical telencephalic structures. Such dedicated brainstem imaging approaches will surely improve the sensitivity and replicability of brainstem neuroimaging studies for pain.

In fact, different neuroimaging techniques are needed to assess brainstem physiology beyond BOLD fMRI. For instance, it has been known for some time that glial cells, which contribute greatly to pain processing,^59^ also influence neurovascular coupling and hence the BOLD fMRI signal.^113^ Thus, future brainstem imaging might also extend beyond “neuroimaging,” using PET ligands sensitive to microglia and astrocyte activity. These techniques hold great promise in exploring glial mechanisms for human pain disorders, and future studies should make greater use of emerging PET techniques with novel, more specific ligands.

Ultimately, although the challenges of brainstem imaging are daunting, recent advances in image acquisition and analysis methods have helped improve the feasibility and robustness of dedicated brainstem imaging research. Furthermore, given the important role that nuclei within this brain region play in the processing of nociception and pain, the coming years should see a notable increase in published neuroimaging research focused on the brainstem.

## Disclosures

The authors have no conflict of interest to declare.
